# Lanthanum Gastropathy in Gastrectomy Specimen: A Case Report

**DOI:** 10.1016/j.gastha.2024.04.001

**Published:** 2024-04-06

**Authors:** Erika M. Dorff, Sarah Y. Liu, Wasef Abu-Jaish, Amer K. Abu Alfa

**Affiliations:** University of Vermont Medical Center, Burlington, Vermont

**Keywords:** Lanthanum Carbonate, End-Stage Renal Disease, Gastropathy, Histiocyte

## Abstract

Lanthanum carbonate (LC) is a phosphate binder used in end-stage renal disease (ESRD) with few adverse effects due to poor systemic absorption. Gastrointestinal deposition is likely due to alterations in epithelial permeability from inflammation in ESRD. It is challenging to detect in cases with minimal deposition and may be missed on endoscopy and biopsy. A 36-year-old with ESRD who was evaluated for gastrectomy was found to have LC deposition histologically. Years later, the excised portion had similar findings. This case allows for evaluation of LC gastropathy in a resection specimen, providing the opportunity to showcase its unique pathology features.

## Introduction

Lanthanum carbonate (LC) is a noncalcium, nonaluminum, oral phosphate binder used in end-stage renal disease (ESRD) patients on dialysis.^1^ It prevents hyperphosphatemia by binding with dietary phosphorus and forming minimally absorbable complexes that are excreted in the stool. Poor absorption of LC lends to its lack of systemic effects.^2^ It has fewer known severe adverse reactions compared to other phosphate binders and may be used as long-term monotherapy for hyperphosphatemia.^3^ Even despite its low bioavailability, some rare gastrointestinal effects linked to LC have been described and include dysphagia, nausea, vomiting, and reflux.^1^

Gastrointestinal deposition of LC was first reported in 2015.^4^^,^^5^ Typical endoscopic findings include gastritis, erosions, ulcerations, and polyps. The gastric mucosa has also been described as having a diffuse white color or fine granular white deposits.^1^ However, detection of these findings may be challenging during endoscopy in cases with minimal deposition. To our knowledge, there have been no reports thus far detailing LC gastropathy in specimens larger than a biopsy or an endoscopic submucosal dissection. We present a case of LC gastric deposition initially discovered on preoperative workup for bariatric surgery, with persistence of these findings several years later in the gastrectomy specimen.

## Case Report

**Figure 1:**
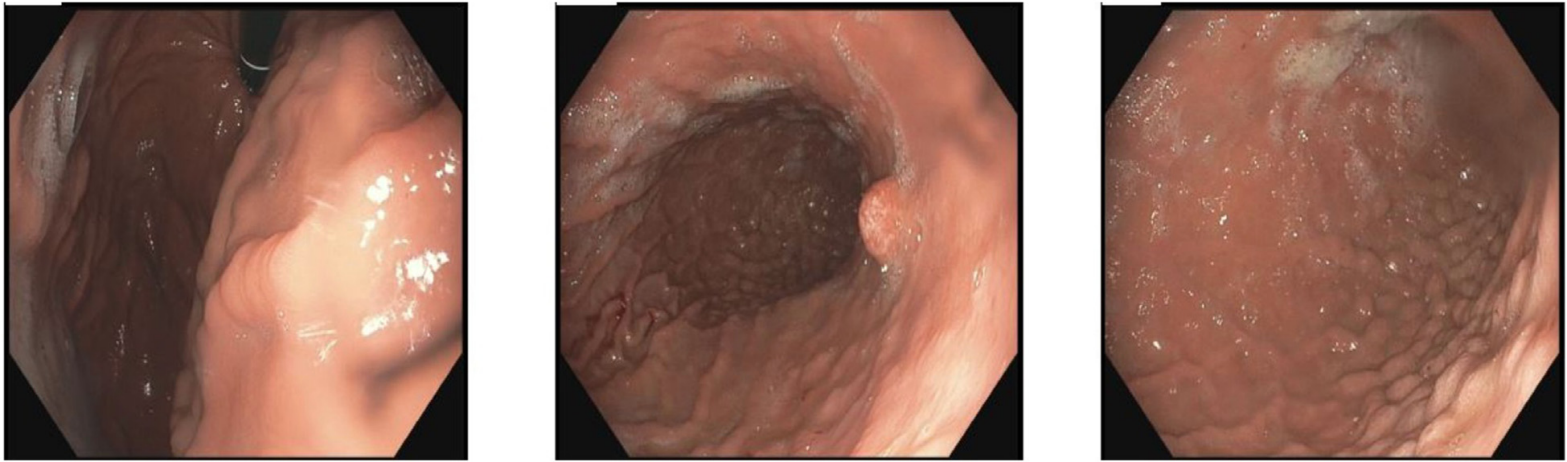
Endoscopic view of gastric mucosa with gastritis and multiple gastric polyps.

The patient is a 36-year-old with history of ESRD secondary to hypertension, nephrosclerosis, and focal segmental glomerulosclerosis likely secondary to obesity related glomerulopathy. She began hemodialysis in 2017. She was started on LC 750 milligrams 3 times daily in July 2017 for hyperphosphatemia, which was titrated up to a maximum dose of 2000 milligrams 3 times daily in February 2018. She was evaluated by bariatric surgery at the University of Vermont Medical Center in July 2019 for sleeve gastrectomy to help lose weight for eventual kidney transplant. An esophagogastroduodenoscopy was completed as part of the preoperative workup. Endoscopic findings were significant for gastritis and multiple gastric polyps (Figure 1). Gastric mucosal and antral polyp biopsies were obtained. Histologically, these demonstrated aggregates of plump histiocytes containing coarse granular eosinophilic material within the superficial lamina propria, consistent with LC gastropathy.

**Figure 2:**
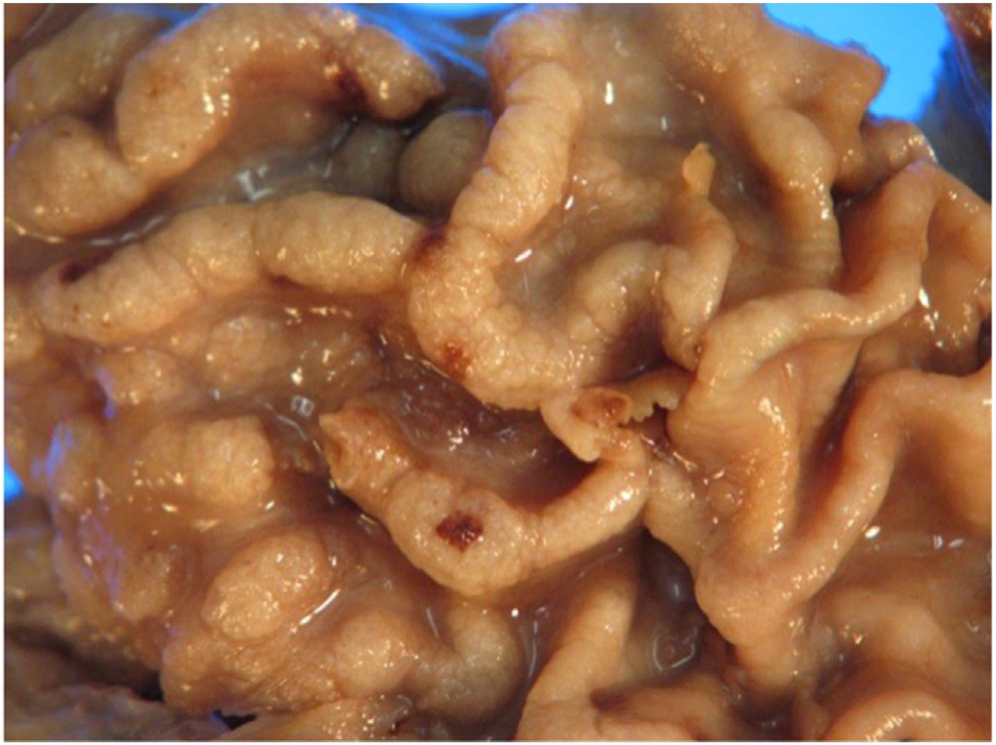
Sleeve gastrectomy specimen showing diffuse mucosal polyposis.

The patient underwent laparoscopic sleeve gastrectomy in October 2022. The gross specimen showed similar features to the 2019 endoscopy: diffuse mucosal polyposis, expanded and bulbous rugal folds with focal erosions (Figure 2).

**Figure 3:**
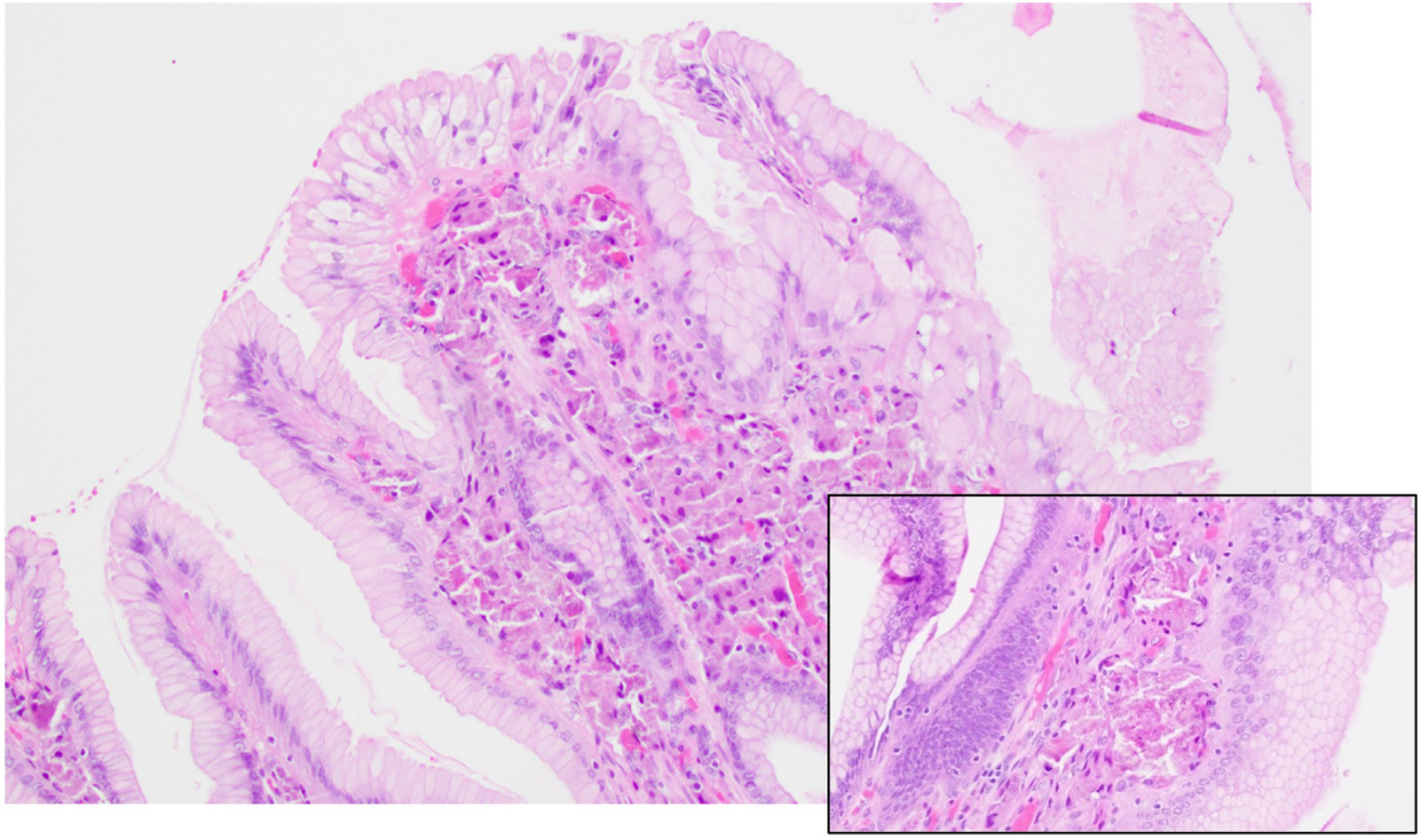
Photomicrograph of gastric mucosa showing polypoid histiocytic aggregates within superficial lamina propria (Hematoxylin and eosin, 20× and 40×).

Histological evaluation demonstrated identical findings to the 2019 biopsies. There were benign hyperplastic-type polyps with eosinophilic histiocytic deposits within the lamina propria, again consistent with LC gastropathy (Figure 3). The histiocytic deposits were limited to the gastric mucosa and were not identified within the submucosa, muscular wall, or serosa. There was no evidence of malignancy.

The patient had been taking LC until approximately 3 weeks prior to surgery. Following sleeve gastrectomy, she was transitioned to sevelamer to treat hyperphosphatemia due to financial reasons. There have been no further studies completed or adverse events reported since the surgery.

## Discussion

To our knowledge, LC gastropathy has not been previously reported in pathology specimens larger than a biopsy or an endoscopic submucosal dissection. Our patient underwent a sleeve gastrectomy for a separate indication and LC gastropathy was an incidental finding, but it allowed for evaluation of the entire gastric wall for the first time. Endoscopic examination can provide a view of the macroscopic gastric mucosa but is limited by the technical skillset of the endoscopist performing the procedure. Subtle changes in the mucosa may be better detected and thoroughly examined in a resection specimen. Our case did not show evidence of LC deposition in deeper layers to the mucosa.

It is difficult to determine the prevalence of LC gastropathy as it is a relatively new medication compared to other phosphate binders. Additionally, not all patient with ESRD taking LC undergo endoscopic and histologic evaluation; prior studies have found deposits in 60%–85% of patients.^6^ There are several proposed mechanisms for LC gastropathy, but the leading hypothesis is that there is an alteration in gastric epithelial permeability due to systemic inflammation in ESRD causing gastric and small intestinal epithelial tight junction disruption.^7^, ^8^, ^9^, ^10^ This allows for the phagocytosis of LC by histiocytes in the gastric mucosa. Shitomi et al also suggested that the alkaline environment within the gastric mucosa contrasted with the luminal acidity would allow crystallization of LC in the lamina propria.^8^

There are no approved treatments for LC gastropathy beyond discontinuation. Additional treatment may not be necessary given lack of systemic effects. As evident by the case presented, it is also unclear how long it persists after discontinuation. Given that it is new compared to other phosphate binders, consequences of its long-term use are relatively unknown, and few studies have examined LC gastropathy prognosis. One case report presented a patient with mucosal LC deposition with concurrent gastric neoplastic changes, although this is the only description of malignant changes in current literature.^11^ The histological findings in this case did not show evidence of neoplasm despite her taking LC for more than 5 years. Further investigation is warranted to evaluate for long-term effects, including complications of deposition and potential association with malignancy.

Lanthanum gastropathy is a relatively underreported phenomenon. Deposition mechanisms are not completely understood, and few theories have been advanced. In this report, we describe endoscopic, gross, and microscopic pathology features as encountered in a gastrectomy specimen. This is the first description of LC gastropathy in a resection specimen, providing the opportunity to increase awareness of this entity. Further studies are justified to evaluate LC gastropathy and potential consequences of its long-term use.
